# Real-World Toxicity and Effectiveness Study of Abemaciclib in Greek Patients with Hormone Receptor-Positive/Human Epidermal Growth Factor Receptor 2-Negative Breast Cancer: A Multi-Institutional Study

**DOI:** 10.3390/cancers17152543

**Published:** 2025-07-31

**Authors:** Elena Fountzilas, Eleni Aravantinou-Fatorou, Katerina Dadouli, Panagiota Economopoulou, Dimitrios Tryfonopoulos, Anastasia Vernadou, Eleftherios Vorrias, Anastasios Vagionas, Adamantia Nikolaidi, Sofia Karageorgopoulou, Anna Koumarianou, Ioannis Boukovinas, Davide Mauri, Stefania Kokkali, Athina Christopoulou, Nikolaos Tsoukalas, Avraam Assi, Nikolaos Spathas, Paris Kosmidis, Angelos Koutras, George Fountzilas, Amanda Psyrri

**Affiliations:** 1 Department of Medical Oncology, St. Luke’s Clinic, 55236 Thessaloniki, Greece; 2 Medical Oncology, European University Cyprus, Engomi 2404, Cyprus; 3 Second Department of Medical Oncology, Metropolitan Hospital, 18547 Piraeus, Greece; elenaravantinou@gmail.com; 4 Hellenic Cooperative Oncology Group, Statistical Department, 11526 Athens, Greece; katerina1dad@gmail.com; 5 Section of Medical Oncology, Department of Internal Medicine, Attikon University Hospital, Faculty of Medicine, National and Kapodistrian University of Athens School of Medicine, 12462 Athens, Greece; giotoik@med.uoa.gr (P.E.); psyrri237@yahoo.com (A.P.); 6 Second Department of Internal Medicine, Agios Savvas Cancer Hospital, 11522 Athens, Greece; tryfonopoulos@hotmail.com; 7 Third Department of Medical Oncology, Hygeia Hospital, 15123 Athens, Greece; nvernadou@oncologists.gr; 8 Department of Medical Oncology, German Oncology Center, Limassol 4108, Cyprus; eleftherios.vorrias@goc.com.cy; 9 Oncology Department, General Hospital of Kavala, 65500 Kavala, Greece; tvagionas@yahoo.com; 10 Oncology Department, Private General Maternity, Gynecological and Pediatric Clinic “MITERA” Hospital, 15123 Athens, Greece; mnikolaidi@mitera.gr; 11 Third Department of Medical Oncology, IASO Clinic, 15123 Athens, Greece; skarageorg@hotmail.com; 12 Hematology-Oncology Unit, Fourth Department of Internal Medicine, Attikon University Hospital, Medical School, National and Kapodistrian University of Athens School of Medicine, 12462 Athens, Greece; akoumari@yahoo.com; 13 Department of Medical Oncology, Theagenio Cancer Hospital, 54639 Thessaloniki, Greece; ibouk@otenet.gr; 14 Faculty of Medicine, School of Health Sciences, University of Ioannina, 45110 Ioannina, Greece; dvd.mauri@gmail.com; 15 Oncology Unit, Department of Internal Medicine, Hippocratio General Hospital, National and Kapodistrian University of Athens School of Medicine, 11527 Athens, Greece; stefikokkali@gmail.com; 16 Medical Oncology Unit, St. Andrew Hospital, 26332 Patras, Greece; athinachristo@hotmail.com; 17 Department of Oncology, 401 General Military Hospital of Athens, 11525 Athens, Greece; tsoukn@yahoo.gr; 18 Second Department of Medical Oncology, Henry Dunant Hospital Center, 11526 Athens, Greece; b.oncology@dunant.gr; 19 Fourth Oncology Department & Comprehensive Clinical Trials Center, Metropolitan Hospital, 18547 Athens, Greece; nikspa2011@gmail.com; 20 Second Department of Medical Oncology, Hygeia Hospital, 15123 Athens, Greece; parkosmi@otenet.gr; 21 Division of Oncology, Department of Medicine, University Hospital of Patras, Medical School, 26504 Patras, Greece; angkoutr@upatras.gr; 22 Laboratory of Molecular Oncology, Hellenic Foundation for Cancer Research, 57001 Thessaloniki, Greece; foutzil@auth.gr; 23 Aristotle University of Thessaloniki, 54124 Thessaloniki, Greece

**Keywords:** abemaciclib, breast cancer, CDK4/6 inhibitor, diarrhea, real-world study, survival, toxicity

## Abstract

Background/Objectives: This study aimed to assess real-world toxicity and efficacy data of patients with early and advanced breast cancer (BC) who received treatment with abemaciclib. Methods: This was a prospective/retrospective multi-institutional collection of clinicopathological, toxicity, and outcome data from patients with early or metastatic hormone receptor (HR)-positive, human epidermal growth factor receptor 2 (HER2)-negative BC who received treatment with abemaciclib in combination with endocrine therapy in departments of oncology in Greece. Treatment combinations of abemaciclib with any endocrine therapy were accepted. The primary end point was toxicity rate in all patients of the study. Results: From June/2021 to May/2024, 245 women received abemaciclib/endocrine combination therapy; the median age was 57 years. Of these, 169 (69%) received abemaciclib as adjuvant therapy for early-stage disease, while 76 (31%) were treated for advanced BC. At the time of the data cutoff, 133 (84.7%) patients remained in the 2-year treatment period. The most common adverse event (AE) was diarrhea (51%), primarily Grade ≤ 2. Dose modifications due to AEs were required in 19.2% of cases, while treatment discontinuation occurred in 5.1%. There was no difference in dose modification/discontinuation rates between older patients (>65 years) and the remaining patients. For early-stage BC patients, the 2-year DFS and OS rates were 90.8% and 100%, respectively. In patients with advanced cancer (70, 30.8%), 1-year PFS and OS rates were 78% and 96.3%, respectively. Conclusions: This study confirms the safety and effectiveness of abemaciclib in alignment with registrational trials offering valuable insights into toxicity management and clinical outcomes in routine practice without identifying new safety concerns. Clinical Trial Registration: ClinicalTrials.gov NCT04985058.

## Simple Summary

This real-world, multi-institutional study assessed the safety and effectiveness of abemaciclib in combination with endocrine therapy for hormone receptor-positive/HER2-negative breast cancer. Our findings confirm the tolerability and efficacy of abemaciclib in both early and advanced settings, including in older patients. Toxicity was manageable with appropriate interventions, while no new safety concerns were reported.

## 1. Introduction

Cyclin-dependent kinases (CDKs) are protein kinases that phosphorylate cellular proteins, leading to their activation or inactivation during the G1 phase of the cell cycle. Highly selective CDK inhibitors (CDKis) block the cyclin D1/CDK 4/6 complex, halting the cell cycle’s progression to the S phase and preventing cancer proliferation. The addition of CDKis to first-line endocrine therapy has significantly improved objective response rates and progression-free survival (PFS) in hormone receptor (HR)-positive, human epidermal growth factor receptor 2 (HER2)-negative advanced breast cancer [1,2,3]. The combination of ribociclib and endocrine therapy as a first-line treatment resulted in significantly improved overall survival (OS) compared to endocrine therapy alone, both in postmenopausal and premenopausal women with HR-positive/HER2-negative advanced breast cancer [4,5].

Recently, the use of CDKis in combination with endocrine treatment has shown significant clinical benefits in high-risk early-stage breast cancer [6,7]. In the monarchE trial, abemaciclib plus endocrine therapy resulted in a 5-year absolute improvement in invasive disease-free survival (DFS) and distant relapse-free survival rates of 7.6% and 6.7%, respectively, compared to endocrine therapy alone [8]. These results led to the approvals of abemaciclib by the U.S. Food and Drug Administration (FDA) and the European Medicines Agency (EMA). In 2024, ribociclib was also approved for early-stage high-risk breast cancer in combination with endocrine treatment, based on the results of the NATALEE clinical trial [6]. Patients who received ribociclib plus an aromatase inhibitor demonstrated improved invasive DFS at 3 years compared to those receiving an aromatase inhibitor alone.

Despite the clear clinical benefits demonstrated in trials from the addition of CDKis to endocrine therapy, it is crucial to evaluate the respective benefits and toxicity profiles in routine clinical practice after drug approval. Real-world evidence has been shown to provide relevant data on efficacy and, more importantly, on toxicity and the management of adverse events (AEs) in patient subgroups that are often underrepresented in clinical trials—such as older patients, those with poor performance status, or those with multiple comorbidities—when conducted according to quality standards [9,10,11]. We have previously reported retrospectively collected real-world toxicity and efficacy data on CDKis (palbociclib and ribociclib) in patients treated at oncology departments affiliated with the HeCOG Group [12]. However, no data on the use of abemaciclib were included, as it had not yet been approved at that time. To date, real-world data on the use of abemaciclib, particularly in early-stage breast cancer, remain limited [13,14].

We designed a prospective/retrospective trial to assess real-world clinical outcomes and AEs in patients with HR-positive, HER2-negative breast cancer treated with abemaciclib and endocrine therapy. Our aim was to evaluate real-world toxicity and efficacy data for patients with breast cancer receiving abemaciclib, with a focus on clinically relevant subgroups.

## 2. Materials and Methods

### 2.1. Patients

This was a prospective/retrospective descriptive analysis of patients with histologically confirmed HR-positive, HER2-negative breast cancer. Patients could be enrolled prospectively by being recorded in the database at the time of abemaciclib initiation, while treatment, outcome, and toxicity data were updated prospectively every six months. In addition, retrospective patient enrollment was also allowed and data entry based on existing patient medical records was permitted. Eligible patients were aged 18 years or older, women of any menopausal status, with HR-positive/HER2-negative breast cancer who had received treatment with abemaciclib in combination with endocrine therapy at HeCOG-affiliated oncology departments. All treatment combinations of abemaciclib with any endocrine therapy were accepted. Patients were included in the analysis if they had received at least one month of treatment with abemaciclib, unless treatment had been discontinued due to AEs.

In October 2021, the FDA approved abemaciclib with endocrine therapy for the adjuvant treatment of adult patients with HR-positive/HER2-negative early breast cancer at high risk of recurrence. The protocol was amended in November 2021, following the EMA approval of abemaciclib in the adjuvant setting, to include patients with early-stage high-risk disease (NCT04985058). Early-stage tumors included non-metastatic, potentially curable breast cancers, in patients undergoing curative-intent surgery, followed by adjuvant treatment [15,16].

### 2.2. Data Collection

A unique identification number was assigned to each patient at the initiation of treatment with abemaciclib in combination with endocrine therapy. This number was linked to each department of oncology in the HeCOG patient database, where all patients who provided consent are recorded for research purposes. Additionally, the patient number was prospectively recorded in a specially formatted HeCOG database (RedCap). This database was specifically designed to meet the study objectives by including detailed fields for clinicopathological, toxicity, and outcome data. Retrospective patient inclusion was also permitted. Pseudoanonymized patient data were recorded in the database, either prospectively or retrospectively, by trained data managers or physicians from the participating centers. Clinical, pathological, treatment, and outcome data were extracted from patient medical records. Pathology data were obtained in detail from histology reports. Toxicity data were collected from medical records based on clinician documentation of reported symptoms and laboratory values during scheduled clinical visits or hospitalizations, if available. Data collection and updates were conducted biannually. The recorded data were evaluated by HeCOG personnel every six months to ensure completeness and quality. This study adhered to the ESMO GROW guidelines for real-world data reporting [15].

All alive patients provided informed consent, while a waiver of consent was obtained for deceased patients. The study was approved by the Institutional Review Board of “Agios Andreas Hospital” (29912/27 July 2021).

### 2.3. Statistical Analysis

The primary endpoint of the study was the toxicity rate in all patients of the study, both in patients with early-stage high-risk and advanced breast cancer. AEs were graded based on Common Terminology Criteria for AEs (CTCAE, version 4.0). Descriptive statistics (counts with percentages for categorical and median values with the corresponding ranges for continuous variables) were used to summarize patient characteristics and other variables of interest. Categorical data were analyzed with the use of the Chi-square test.

Secondary endpoints of interest included the assessment of disease-free survival (DFS), defined as the time interval from the initiation of treatment to the date of local, regional, distant recurrence, or death from any cause or last contact, whichever occurred firs; PFS, defined as the time interval from the initiation of treatment to the date of first documented progression or death from any cause or last contact, whichever occurred first; and OS, defined as the time interval from the initiation of treatment to death from any cause or last contact. Patients alive were censored at the date of last contact.

Survival curves were estimated using the Kaplan–Meier method and compared across groups with the log-rank test. Significance was set at 5% and all tests were two-sided. Analysis was performed using R language (version 4.3.1) (R Core Team: R: A Language and Environment for Statistical Computing Vienna, Austria: Foundation for Statistical Computing. Available from: http://www.R-project.org/, accessed on 1 June 2025). Ggplot2 (version 4.3.1), survminer, and survival packages were employed to conduct survival analysis and present the Kaplan–Meier curves.

## 3. Results

### 3.1. Patient Characteristics

From June 2021 to June 2024, 245 women received combination treatment with abemaciclib and endocrine therapy; the median age was 57 years, with 167 (68.2%) patients being postmenopausal. Abemaciclib was administered as adjuvant treatment in 169 (69.0%) patients with early-stage high-risk cancer and as palliative treatment in 76 (31.0%) patients with advanced cancer. In both the early-stage and advanced disease groups, aromatase inhibitors were the most commonly used agents (86.4% and 68.4%, respectively). Among patients with advanced disease, abemaciclib was administered as first-line treatment in 57 of 76 (75%) patients, second-line in 12 (15.8%), and third-line or beyond in 7 (9.2%) patients. In patients with advanced cancer, a median of one site of metastasis was reported at the diagnosis of advanced disease, most commonly involving bone lesions (28 patients, 36.8%). Late prescription renewals were uncommon in both groups, with only four patients (1.7%) arriving late for their prescription. Detailed patient characteristics are reported in Table 1.

### 3.2. Toxicity

Toxicity rates were analyzed and reported for all patients in the study. The most common AE was diarrhea, occurring in 51.0% of patients. While the majority of cases were Grade 1 or Grade 2 (44.9%), some patients reported Grade 3 (5.3%) or Grade 4 (0.4%) diarrhea. The onset of diarrhea was reported at a median of 7 days after treatment initiation (range: 1 to 270 days). Patients received loperamide at a median of 1 day from the onset of diarrhea, and the median duration of diarrhea was 6 days. Dose reduction occurred in 47 patients (19.2%) by 1 level and in 6 patients (2.5%) by 2 levels. Recurrence of diarrhea was reported in 35 patients (14.3%), and only 2 patients (0.8%) were hospitalized due to diarrhea. The majority of patients (98.6%) had been educated about the potential for diarrhea and had received prophylactic measures, including dietary adjustments (86.2%).

Other common AEs included fatigue (17.6%), arthralgia (6.9%), leukopenia (6.1%), and nausea (5.3%). No thrombotic events were reported. AEs led to dose modifications in 42 patients (26.8%) and treatment discontinuations in 8 patients (5.1%). Hospitalizations due to AEs were infrequent, occurring in 5.4% of early-stage and 7.0% of advanced disease patients. No deaths associated with AEs were reported in this group.

There was no significant difference in Grade 3/4 toxicity rates (8.9% vs. 10.5%, p = 0.863), dose reduction rates (28% vs. 23.7%, *p* = 0.482), or discontinuation rates due to toxicity (6.0% vs. 6.6%, *p* = 0.775) between patients with early-stage disease compared to those with advanced disease.

**Figure 1:**
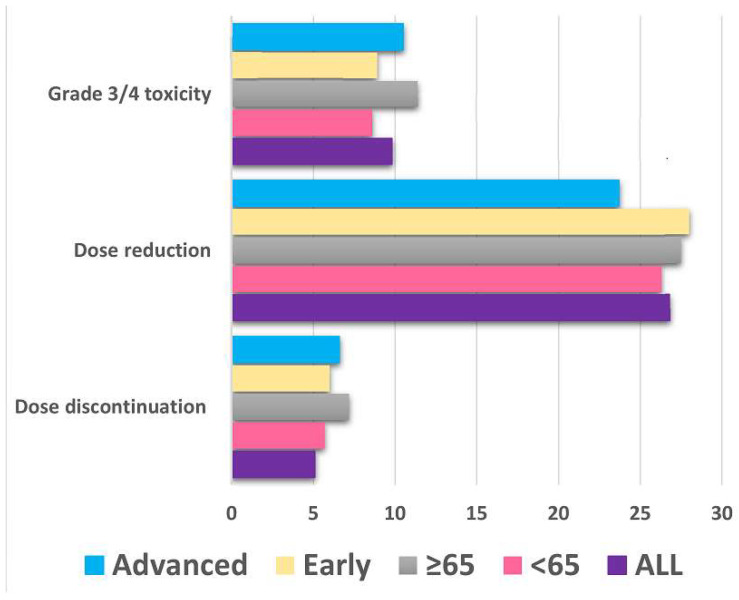
Grade 3/4 toxicity, dose reduction, and discontinuation rates in patients receiving treatment in the early or advanced setting and according to age at the time of treatment with abemaciclib. Dose discontinuation reported was associated with toxicity based on patient preference and physician decision.

Similarly, there was no significant difference in Grade 3/4 toxicity (8.6% vs. 11.4%, *p* = 0.489), dose reduction (26.3% vs. 27.5%, *p* = 0.842), or discontinuation rates from toxicity (5.7% vs. 7.2%, *p* = 0.561) between patients younger than 65 years and those aged 65 years or older. Table 2 summarizes the AEs observed in the study population, highlighting both their overall incidence and severity by grade. Dose reduction, dose interruption, and discontinuation rates among the study patients are depicted in Figure 1.

### 3.3. Clinical Outcomes

#### 3.3.1. Patients in Early-Stage High-Risk Breast Cancer

**Figure 2:**
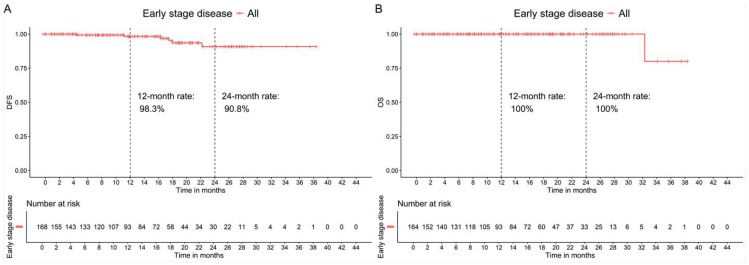

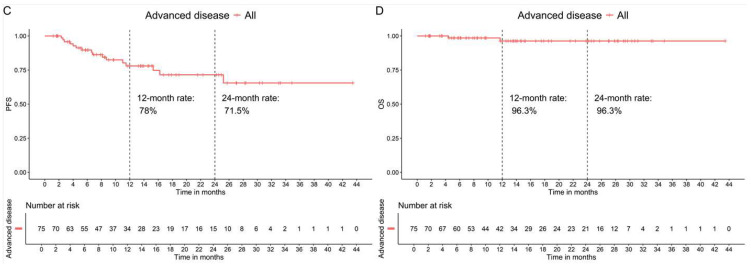
Clinical outcomes in patients with early-stage high-risk breast cancer (*n* = 169) who received adjuvant treatment with abemaciclib in combination with endocrine treatment. (**A**) Disease-free survival (DFS). (**B**) Overall survival (OS). Clinical outcomes in patients with advanced breast cancer (*n* = 76) who received treatment with abemaciclib in combination with endocrine treatment as any line of treatment. (**C**) Progression-free survival (PFS) and (**D**) OS.

At the time of data cutoff (June 2024), 4 patients (2.5%) had completed the 2-year treatment period, while 133 patients (84.7%) remained in the treatment period. With a median follow-up of 11.8 months, six patients experienced disease recurrence, resulting in 2-year DFS and OS rates of 90.8% and 100%, respectively (Figure 2A,B).

#### 3.3.2. Patients with Advanced Cancer

With a median follow-up of 11.8 months, 16 patients progressed while receiving treatment with abemaciclib in combination with endocrine therapy. Clinical benefit was reported for 59 patients (77.6%), with 32 patients (42.1%) demonstrating partial or complete response, while the remaining 27 patients (35.5%) had stable disease. Additionally, 12 patients had non-evaluable disease (15.8%). Overall, five patients (6.6%) experienced disease progression at the first evaluation. Only two patients (2.6%) had a relapse in the central nervous system after treatment with abemaciclib and endocrine therapy. The 1-year progression-free survival (PFS) and OS rates were 78% and 96.3%, respectively (Figure 2C,D).

**Figure 3:**
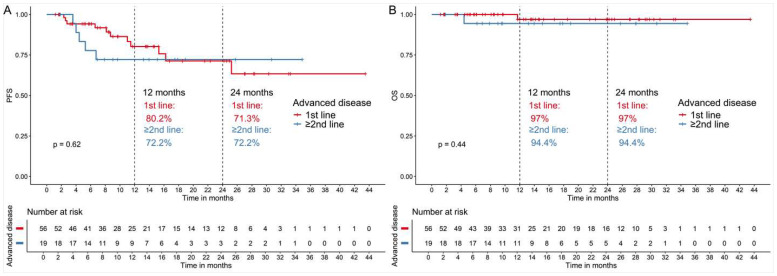
Clinical outcomes between patients with advanced breast cancer who received treatment with abemaciclib in combination with endocrine treatment as first-line of treatment vs. second-line and beyond. (**A**) Progression-free survival (PFS) and (**B**) overall survival (OS).

There was no difference in PFS or OS between patients who received abemaciclib as part of first- or second-line treatment and those treated beyond these lines (Figure 3).

## 4. Discussion

This study provides real-world data on the use of abemaciclib in combination with endocrine therapy in Greek patients with HR-positive, HER2-negative breast cancer, both in early and advanced settings. Our findings confirm the safety and efficacy of abemaciclib in an unselected patient population, consistent with results from the pivotal MONARCH clinical trials. The observed toxicity profile aligns with previous reports, with diarrhea being the most frequently reported AE. Importantly, the majority of patients experiencing diarrhea reported manageable events with supportive care, while severe diarrhea was infrequent. Dose reduction and discontinuation rates in the total population and in clinically relevant subgroups were within expected ranges, further supporting the tolerability of abemaciclib in a real-world setting.

Previous randomized clinical trials have demonstrated improved clinical outcomes with the combination of CDKi and endocrine therapy compared to endocrine therapy alone in patients with metastatic HR-positive/HER2 negative breast cancer [1,2,3,4,5]. Importantly, clinical benefit has been demonstrated both in endocrine-sensitive or -resistant patients, as well as premenopausal or postmenopausal patients. These studies have established this combination as standard-of-care first-line treatment in this clinical setting [16,17]. CDKis have also been evaluated as monotherapy in patients with HR-positive/HER2-negative disease. In the TREnd trial, palbociclib demonstrated moderate clinical activity as a single agent compared to its combination with endocrine therapy [18]. Similarly, in the next MONARCH trial, abemaciclib demonstrated clinical activity as monotherapy in endocrine refractory metastatic breast cancer [19]. Monotherapy with abemaciclib has been approved for patients with HR-positive/HER2-negative metastatic breast cancer with disease progression following endocrine therapy and prior to chemotherapy in the metastatic setting.

In addition, several real-world studies have previously assessed the safety and efficacy of CDK4/6 inhibitors, including palbociclib [20,21] and ribociclib [22]. Selected clinical trials have reported clinical outcomes for all three CDK4/6 inhibitors [23,24,25,26,27]. However, data on abemaciclib in routine clinical practice are limited [23,24]. In our study, efficacy outcomes were within expected ranges. However, since follow-up was relatively short, especially for early-stage patients, a longer follow-up is warranted to determine a clinically relevant benefit from abemaciclib treatment. Our study evaluated survival endpoints, including OS and DFS or PFS as primary effectiveness outcomes, that are considered clinically relevant, and most importantly, more reliable compared to others (i.e., treatment response or quality of life assessments) that are not as accurately captured from routine healthcare data sources. Future real-world studies integrating patient-reported outcomes may provide a more comprehensive assessment of effectiveness beyond survival outcomes alone (ENDURANCE trial, NCT04985058).

In terms of toxicity, previously published observational trials report on toxicity and discontinuation rates [13,14,28]. In a retrospective observational study involving 374 patients who received adjuvant therapy with abemaciclib in Japan, dose reductions were observed in 42.0% patients [14]. Racial and ethnic genetic differences may be associated with increased toxicity rates [29]. In another study, AEs led to discontinuation of abemaciclib in 18.8% of patients in the adjuvant setting and 57.1% in the metastatic setting; however, this analysis involved only 30 patients [13]. A study of 469 patients receiving adjuvant abemaciclib reported a discontinuation rate of 21.5% [28]. Interestingly, investigators noted significantly lower rates of abemaciclib prescribing in 2021, which increased over time. These trials underscore the need to educate physicians and patients about the clinical value and toxicity profile of abemaciclib, as well as to address barriers to its optimal use and AE management. In our study, discontinuation rates were lower than previously reported. Additionally, there was no difference in toxicity and discontinuation rates between older and younger patients. The optimal dose for older patients often becomes a critical issue in clinical practice [30]. It is important to consider that elderly patients treated with abemaciclib were fit, making them suitable candidates for the treatment. Ongoing clinical trials are evaluating whether frail older patients need to receive lower doses of CDKi, including abemaciclib, to limit serious AEs without compromising efficacy (NCT06044623) [31]. Finally, toxicity issues may arise as innovative combinations with CDKis are evaluated in diverse settings [32,33].

Our study did not reveal significant differences in severe toxicity or discontinuation rates between patients with early-stage disease compared to those with advanced disease. In general the non-overlapping mechanisms of toxicity of endocrine therapy and abemaciclib have enabled the safe administration of combination treatment. In addition, proactive management of potential adverse events, particularly diarrhea, has been shown to effectively minimize toxicity and treatment interruptions [34]. These efforts include early patient education on potential adverse events and prompt initiation of supportive measures, including the administration of loperamide and dietary modifications. Importantly, in our study, the majority of the patients, both with early-stage and advanced disease had be informed about the possibility of diarrhea and had been instructed accordingly. Finally, ongoing close patient monitoring, timely dose adjustments, and individualized patient management may have contributed to the safe administration of abemaciclib and endocrine treatment in the majority of patients, regardless of disease stage. The value of digital support tools personalized based on adverse events reported through specially designed digital platforms is being evaluated in a clinical trial (NCT04985058).

Despite its strengths, this study has certain limitations. First, its partially retrospective nature may introduce selection bias and variability in treatment practices across institutions. Additionally, while measures were taken to ensure high-quality data collection and frequent updates of the database, the study’s retrospective nature may have also introduced information bias. Indeed, toxicity rates may be lower compared to those reported in clinical trials, as Grade 1/2 AEs may sometimes be overlooked in patients’ medical records. However, serious Grade 3/4 toxicities are rarely misreported and are therefore captured in real-world studies. Furthermore, the relatively small sample size limited analysis within different subgroups in terms of statistical power. Finally, since no patients that had received palbociclib or ribociclib were included, no direct comparisons could be made to guide informed clinical decision-making in this setting. Future research on the individualization of CDKi selection would provide clinically relevant data [35].

## 5. Conclusions

Our findings contribute to the growing body of evidence by demonstrating that abemaciclib is well tolerated among various patient subgroups, including older individuals and those with comorbidities. Future studies should focus on identifying predictive biomarkers, refining treatment strategies, and expanding real-world evidence to enhance patient outcomes.

## Figures and Tables

**Table 1:** Patient characteristics.

	Early-Stage (*n* = 169)	Advanced (*n* = 76)	Total (*n* = 245)
Age at diagnosis Median [IQR]	56.0 [16.0]	59.5 [21.3]	57.0 [18.0]
The patient comes from urban centers, *n* = 244	115 (68.5%)	42 (55.3%)	157 (64.3%)
Previous history of other cancers, *n* = 244	8 (4.8%)	3 (3.9%)	11 (4.5%)
Family history of breast or ovarian cancer, *n* = 237	38 (23.3%)	15 (20.3%)	53 (22.4%)
Family history of other cancers, *n* = 234	54 (34.0%)	23 (31.1%)	77 (32.9%)
Location, *n =* 243			
Bilateral	8 (4.7%)	4 (5.4%)	12 (4.9%)
Left breast	83 (49.1%)	38 (51.4%)	121 (49.8%)
Right breast	78 (46.2%)	32 (43.2%)	110 (45.3%)
Tumor size, *n* = 194			
T1 0–2 cm	44 (29.3%)	18 (40.9%)	62 (32.0%)
T2 2.1–5 cm	72 (48.0%)	15 (34.1%)	87 (44.8%)
T3 ≥ 5 cm	34 (22.7%)	11 (25.0%)	45 (23.2%)
Histology grade, *n = 239*			
1	8 (4.7%)	3 (4.3%)	11 (4.6%)
2	105 (62.1%)	43 (61.4%)	148 (61.9%)
3	56 (33.1%)	24 (34.3%)	80 (33.5%)
LVI/PNI, *n =* 208	78 (50.3%)	26 (49.1%)	104 (50.0%)
Nodal status, *n* = 209			
0	9 (5.4%)	15 (35.7%)	24 (11.5%)
1–3	43 (25.7%)	14 (33.3%)	57 (27.3%)
≥4	115 (68.9%)	13 (31.0%)	128 (61.2%)
Estrogen Receptor status, *n* = 245			
ER-positive	169 (100%)	76 (100%)	245 (100%)
ER positivity value (%)	95.0 [15.0]	95.0 [20.0]	95.0 [15.0]
Progesterone Receptor status, *n = 245*			
Positive	144 (85.2%)	61 (80.3%)	203 (83.5%)
PR positivity value (%)	80.0 [55.0]	70.0 [62.0]	80.0 [55.0]
Ki67 (%)	20.0 [15.0]	20.0 [16.5]	20.0 [15.0]
Genetic testing, result, *n = 86*			
Mutation	16 (23.9%)	4 (23.5%)	20 (23.8%)
Negative	47 (70.1%)	13 (76.5%)	60 (71.4%)
VUS	4 (6.0%)	0 (0.0%)	4 (4.8%)
If mutation, specify #			
BRCA1	5 (31.3%)	0 (0.0%)	5 (25.0%)
BRCA2	3 (18.8%)	1 (25.0%)	4 (20.0%)
CHEK2	1 (6.3%)	1 (25.0%)	2 (10.0%)
PALB2	3 (18.8%)	0 (0.0%)	3 (15.0%)
ATM	4 (25.0%)	0 (0.0%)	4 (20.0%)
OTHER	3 (18.8%)	2 (50.0%)	5 (25.0%)
Menopausal status (*n* = 236)			
Premenopausal/Perimenopausal	50 (30.9%)	19 (25.7%)	69 (29.2.0%)
Postmenopausal	112 (69.1%)	55 (74.3%)	167 (70.8%)
Chemotherapy adjuvant/neoadjuvant	163 (96.4%)	ΝA	
Endocrine therapy (*n* = 245)	169 (100%)	76 (100%)	
Tamoxifen	18 (10.8%)	5 (6.6%)	
Aromatase inhibitor	150 (88.8%)	52 (68.4%)	
Fulvestrant	1 (0.6%)	20 (26.3%)	
De novo metastatic disease		40 (52.6%)	
Site of metastasis at diagnosis			
Locoregional (axillary and supraclavicular nodes, skin, breast)		18 (23.7%)	
Bones		28 (36.8%)	
Lung		12 (15.8%)	
Liver		3 (3.9%)	
Other *		14	

Abbreviations: * Brain, other breast, bone marrow, adrenal, intestine, leptomeningeal dissemination, peritoneum, muscle. # Selected patients had germline mutations in more than 1 gene.

**Table 2:** Adverse events.

Adverse Events	*n* (%)	Grade			
		1	2	3	4
Diarrhea	125 (51.0%)	64 (26.1%)	46 (18.8%)	13 (5.3%)	1 (0.4%)
Fatigue	43 (17.6%)	31 (12.7%)	10 (4.1%)	1 (0.4%)	
Arthralgia	17 (6.9%)	13 (5.3%)	4 (1.6%)		
Leukopenia	15 (6.1%)	8 (3.3%)	6 (2.4%)	1 (0.4%)	
Nausea	13 (5.3%)	10 (4.1%)	3 (1.2%)		
Anemia	13 (5.3%)	7 (2.9%)	5 (2.0%)	1 (0.4%)	
ALT/AST increased	12 (4.9%)	9 (3.7%)	1 (0.4%)	2 (0.8%)	
Neutropenia	11 (4.5%)	6 (2.4%)	3 (1.2%)	2 (0.8%)	
Abdominal Pain	10 (4.1%)	8 (3.3%)	1 (0.4%)		
Stomatitis/Dry Mouth	9 (3.7%)	6 (2.4%)	2 (0.8%)		
Anorexia	9 (3.7%)	6 (2.4%)	3 (1.2%)		
Headache	6 (2.4%)	6 (2.4%)			
Dyspepsia	6 (2.4%)	2 (0.8%)	4 (1.6%)		
Hot Flashes/night sweats	5 (2.0%)	5 (2.0%)			
Vomiting	5 (2.0%)	5 (2.0%)			
Skin Rash	4 (1.6%)	2 (0.8%)	1 (0.4%)		
Dry skin	4 (1.6%)	2 (0.8%)	1 (0.4%)		
Constipation	3 (1.2%)	2 (0.8%)	1 (0.4%)		
Infection	3 (1.2%)	2 (0.8%)		1 (0.4%)	
Changes in food taste	3 (1.2%)	1 (0.4%)	2 (0.8%)		
Depression	3 (1.2%)	1 (0.4%)	2 (0.8%)		
Pruritus	2 (0.8%)	2 (0.8%)			
Vaginal dryness	2 (0.8%)	2 (0.8%)			
Dizziness	2 (0.8%)	2 (0.8%)			
Insomnia	2 (0.8%)	2 (0.8%)			
Thrombocytopenia	1 (0.4%)	1 (0.4%)			
Other *	11 (4.5%)	7 (2.9%)	2 (0.8%)	2 (0.8%)	

* Grade 3: hyponatremia, edema, Grade 2: dyspnea, renal dysfunction, hypokalemia.

## Data Availability

The data underlying this article are available in the article and in its online additional material.

## Abbreviations

The following abbreviations are used in this manuscript:

AE	adverse event
BC	advanced breast cancer
CDK	cyclin-dependent kinases
DFS	disease-free survival
EMA	European Medicines Agency
FDA	U.S. Food and Drug Administration
HeCOG	Hellenic Cooperative Oncology Group
HER2	human epidermal growth factor receptor 2
HR	hormone receptor
OS	overall survival
PFS	progression-free survival
